# Cross-Cultural Examination of Problematic Internet Use and Associated Psychological Variables: A Comparative Study in Italy, Spain, Ecuador, and Peru

**DOI:** 10.3390/jcm13123451

**Published:** 2024-06-13

**Authors:** Manuel Varchetta, Ginevra Tagliaferri, Emanuela Mari, Alessandro Quaglieri, Clarissa Cricenti, Manuel Martí-Vilar

**Affiliations:** 1 Department of Basic Psychology, Faculty of Psychology and Speech Therapy, University of Valencia, Av. Blasco Ibañez, 21, 46010 Valencia, Spain; varchett@alumni.uv.es; 2 Department of Psychology, Sapienza University of Rome, Via dei Marsi 78, 00185 Rome, Italy; ginevra.tagliaferri@uniroma1.it (G.T.); e.mari@uniroma1.it (E.M.); alessandro.quaglieri@uniroma1.it (A.Q.); clarissa.cricenti@uniroma1.it (C.C.); 3 Faculty of Social and Communication Sciences, Universitas Mercatorum, Piazza Mattei 10, 00186 Rome, Italy

**Keywords:** cross-cultural internet use, problematic internet use, internet addiction, social media addiction, internet gaming disorder, FoMO, phubbing

## Abstract

**Background:** Several studies focused on the escalating prevalence of Problematic Use of Internet (PUI) and its consequential impact on mental health globally. This study investigates the relationship between PUI and associated psychological variables across different cultural contexts in Italy, Spain, Ecuador, and Peru. **Method:** A total of 675 participants, aged 18 to 54 (M = 22.73; SD = 4.05), completed measures assessing Internet addiction, social media addiction, Fear of Missing Out, Internet Gaming Disorder, and Phubbing. **Results:** Significant cultural variations were found, with Italian participants showing higher levels of Internet addiction but lower levels of social media addiction compared to other countries. Fear of Missing Out was higher in Italy, while the Italian sample exhibited lower Internet Gaming Disorder levels compared to Peru. As regards the communication disturbance caused by Phubbing, the Italian sample demonstrated significantly higher scores than the Peruvian sample. Linear regression analyses revealed distinct predictors for problematic Internet use in each country, emphasizing the importance of considering the cultural context in understanding this phenomenon. **Conclusions:** These findings contribute valuable insights into the interplay of cultural factors, psychological variables, and problematic Internet use, guiding future research and interventions.

## 1. Introduction

Problematic Use of Internet (PUI) has been defined as “the inability to control Internet use that leads to negative consequences in daily life” [1]. Although Internet use has increased exponentially in recent years, the scientific literature remains divided over the terminology used to describe Internet use and abuse [2]. The debate centers on whether the terms “Internet addiction (IA)” and PUI can be differentiated or considered as equivalent. In 1998, Young [3] defined “Internet addiction (IA)” to encompass a variety of network-related behaviors and impulse control disorders: compulsive online gambling, cyber addiction, multiuser dimensions (MUD), information overload addiction, Internet Gaming Disorder (IGD), and cyber addiction, combined with social media addiction (SMA), leading to a distorted perception of virtual versus real relationships and causing significant interpersonal and family problems. Both IGD and SMA are largely studied in the scientific literature [4].

SMA is defined as “the inability to regulate the use of social networks, resulting in negative personal and interpersonal effects” [5], and is manifested through the constant monitoring of accounts (Instagram, Facebook, Twitter). IGD is the only network-related addiction currently included in the latest version of the International Classification of Diseases (ICD-11) in the “disorder due to addictive behavior” section [4]. Moreover, it is proposed as a condition included in the “conditions for future research” section of the DSM-5 TR [6]. Gambling disorder, predominantly online, is characterized by a persistent or recurrent pattern of gaming behavior (“digital gaming” or “video gaming”) and it is associated with negative consequences (e.g., social, work, family, educational) as well as functional impairment, as recognized by the World Health Organization [7]. Both SMA and IGD are linked to specific personological characteristics, including impulsivity, social withdrawal, reduced social and empathic skills, difficulties in emotion regulation, and attention problems, suggesting a bidirectional influence on excessive gaming [8,9].

Two of the most well-known phenomena in the context of problematic Internet use are Phubbing and Fear of Missing Out (FoMO) [10]. Phubbing, defined as the act of looking at a smartphone while having a face-to-face conversation with others [11], is influenced by factors such as excessive Internet use, technology addictions (social media, smartphones, etc.), lack of self-control, social anxiety, and anxiety traits [12]. Studies have shown a strong correlation between Phubbing and PUI, as individuals who frequently engage in Phubbing behaviors often struggle with various forms of Internet and technology addiction [13,14]. Additionally, Phubbing can exacerbate feelings of social exclusion and loneliness, reinforcing a cycle of technology dependence and social withdrawal [15].

FoMO is identified as a form of social anxiety characterized by the desire to stay continuously connected with what others are doing and the fear of being excluded from rewarding events, experiences, or social contexts [10]. FoMO has been recognized as a significant predictor of problematic Internet use and technology addiction, by noticing that individuals with high levels of FoMO are more likely to engage in excessive online activities to avoid missing out on social interactions and updates [16,17,18]. This makes FoMO a critical variable to explore in the context of PUI, as it directly influences individuals’ online behaviors and their susceptibility to Internet-related problems.

Epidemiological data on PUI and technological addictions in the countries involved in the study can provide valuable context for understanding the significance of these issues. Recent studies indicate that Internet addiction rates in Italy range from 8% to 12% among young adults, with significant concerns about social media addiction [19]. In Spain, research from 2022 suggests that around 8.1% of adolescents exhibit problematic Internet use, with notable increases in social media and smartphone addiction [20]. However, due to the complexity of the phenomenon and the methodological and cultural heterogeneity, it remains difficult to make an exact estimate of the prevalence of PUI. Indeed, little data exist on PUI prevalence rates among South American Countries. Most of the epidemiological studies on PUI have been conducted in Europe, where the behavior seems to be widespread, especially among adolescents in a percentage ranging between 5% and 15.2%, and in Asian countries, where the rate of young people with problems linked to Internet use ranges between 2.5% and 26.8 [21,22]. Hence, it is highly important to investigate PUI and related factors across diverse cultural contexts to inform effective prevention and intervention strategies.

## 2. Internet Overuse and Personality

Several studies [23] suggest specific personality traits, emotional dysregulation, prosociality, the Big Five, and impulsivity to be underlying factors of problematic Internet use.

Gratz and Roemer [23] conceptualized emotion regulation as the awareness, understanding, and acceptance of emotions; the ability to control impulsive behaviors; and the use of appropriate regulation strategies to satisfy individual goals and situational demands. According to Gratz and Roemer [23], the absence of one or more of these skills might indicate the presence of emotion dysregulation problems. Furthermore, high levels of emotional dysregulation are associated with problematic Internet use, video game addiction, and excessive use of social media [24]. People with emotional dysregulation often experience difficulties in developing healthy relationships due to maladaptive coping strategies, whereby the Internet may become a means to gain more self-control and improve communication within relationships [25]. This is also consistent with findings by Varchetta and colleagues [26], showing that the FoMO mediates the relationship between emotional dysregulation and excessive Internet use.

Prosocial behavior is conceptualized as the set of acts/behaviors defined by society and/or one’s social group as “generally beneficial to other people” [27]. Prosociality has been extensively investigated in the literature [28], and it is associated, for example, with a better school performance [29], higher self-esteem [30] and subjective well-being, as well as positive affective states [31] in offline contexts. However, this construct has been less researched within the online context, which differs in its dynamics and characteristics (e.g., absence of face-to-face feedback). Indeed, in this context, typical prosocial behaviors can be enacted on social networks, e.g., by creating “groups” to give and receive support from informal networks of peers as well as from strangers [32]—or on gaming platforms, where people can help each other during gaming activities [33]. Some studies [34] also show a negative correlation between prosocial behaviors and excessive Internet use.

The “Big Five” theory (i.e., extroversion, agreeableness, conscientiousness, neuroticism, and openness to experience) [35] represents the most widely accepted theory in explaining human behavior in several domains, including the tendency to abuse or not to abuse the Internet [24]. More specifically, low scores in conscientiousness and extraversion indicate difficulties in pursuing one’s goals, self-discipline, and engaging in stable social relationships, while high scores in neuroticism indicate increased nervousness, irritability, moodiness, insecurity, somatization, sadness, and melancholy [36]. This is in line with the findings that have emerged in the literature [36] that underline how conscientiousness, agreeableness, and extroversion are negatively associated with excessive Internet behaviors, such as excessive social media use and IGD, while neuroticism correlates positively with these behaviors.

Finally, impulsivity is a key trait in the development and maintenance of addictions by affecting the decision-making abilities of individuals, causing an overestimation of immediate rewards and/or a reduced evaluation of long-term rewards [37]. Indeed, studies have shown that the reward circuitry is one of the neurocognitive aspects most implicated in both substance and behavioral addictions [38], including excessive Internet use and IGD [39].

## 3. The Influence of Context on Internet Overuse

The widespread use of the Internet and related technologies has led to an increase in their overuse globally. However, specific contexts and cultures would seem to influence behaviors that can lead to the development of addiction [40]. Social norms and national cultural values, such as risk propensity, level of uncertainty avoidance, preference for face-to-face interaction, concept of time, and gender dynamics, have been identified as factors that can facilitate or hinder the adoption of information technologies, change how they are used, and affect the outcomes of such use [41].

For example, concerning Internet use, Nath and Murthy’s [42] study indicates that specific cultural traits, such as uncertainty, avoidance, and masculinity, have a significant impact on Internet use in different nations. Not only does culture play a role in the enactment of behaviors, but it is also strongly linked to the expression of different personality characteristics. A study by Schmitt and colleagues [43] of 56 nations showed that South American and European countries tend to score higher on the dimension of openness, with Chile ranking first among all cultures. However, in relation to other dimensions, for instance extraversion, South America, along with Southeast Asia, tends to show significantly lower scores than Europe.

Regarding the relationship between excessive Internet use and personality variables, it has been found that addictive behaviors, such as social media addiction or video game addiction, and other dysfunctional behaviors related to the digital context (e.g., Phubbing), are significantly predicted by emotional dysregulation, low perception of psychological need satisfaction, low self-esteem, specific Big Five personality traits and FoMo in the European context [17,24]. Similarly, among the South American population, FoMo is a significant predictor of forms of technological dependence and behaviors such as Phubbing [44]. Nevertheless, personality factors associated with Internet use have not been particularly explored in this context, but rather behavioral patterns within the digital context, such as immersion, desire to influence others, loneliness, and feelings of belonging [45].

Moreover, several studies highlight the influence of cultural factors on problematic Internet use across different countries. In the United States, where both high Internet and social media use are prevalent, research has shown a correlation with psychological issues such as depression, anxiety, and impulsivity. In particular, American youth exhibit vulnerability to social media addiction and Fear of Missing Out (FoMO), resulting in higher Internet Addiction Test (IAT) scores and emotional dysregulation [46,47]. Similarly, in South Korea, there is a notable prevalence of gaming addiction, exacerbated by cultural pressures for academic and professional success, leading to stress and escapism behaviors [48].

In China, factors such as the one-child policy and intense academic competition contribute to high Internet use among adolescents. Internet addiction has been recognized as a significant health issue, with studies indicating associations with loneliness, family dynamics, and impulsivity, prompting governmental measures to address excessive gaming [49,50]. Likewise, in Japan, a combination of traditional and modern influences has resulted in significant Internet and gaming addiction, often accompanied by social withdrawal and mental health challenges, such as depression and anxiety. Elevated scores on the Internet Addiction Test have been linked to emotional issues and introverted behavior [51,52]. These findings underscore the critical necessity of exploring cross-cultural disparities in the development of problematic Internet use. Therefore, our research aims to delve into the influence of cultural differences on Internet-related disorders, providing valuable insights for a deeper understanding of these phenomena across diverse cultural contexts.

## 4. Research Questions

RQ1: To investigate the presence of possible differences in technological (i.e., Internet addiction, social media addiction, social media engagement, gaming addiction, Phubbing, Fear of Missing Out) and personological (difficulties in emotion regulation, personality traits, prosociality, and impulsivity) variables across countries (i.e., Italy, Spain, Ecuador, and Peru).

RQ2: To investigate the presence of possible relationships between Internet addiction and technological (i.e., Internet addiction, social media addiction, social media engagement, gaming addiction, Phubbing, Fear of Missing Out) and personological (difficulties in emotion regulation, personality traits, prosociality, and impulsivity) variables depending on countries (i.e., Italy, Spain, Ecuador, and Peru).

## 5. Participants

A total of 862 participants were initially involved in the study, using a non-probabilistic and convenience sampling technique. The inclusion criteria required participants to be of legal age (18 years) and residents of one of the four countries identified for the study (Italy, Spain, Ecuador, and Peru). Of the 862 participants, 8 did not provide their consent, and 179 did not complete the entire questionnaire. Consequently, the final sample consisted of 675 participants. Only fully completed questionnaires were included to ensure the integrity and reliability of the data, resulting in the exclusion of incomplete responses from the analysis. The final sample comprised 251 males and 424 females, with ages ranging from 18 to 54 years, (M = 22.73, SD = 4.05).

The sample composition is described below:

Italian sample: 186 participants (86 males and 100 females) with an age range from 18 and 30 (M = 22.15, SD = 3.29). Among these participants, 66.7% had secondary education, 19.4% had a graduate degree, 9.7% had a master’s or doctoral degree, and 2.7% had only primary education. Additionally, 80.6% were students, 80.6% lived with their families, and 52.7% had a partner.

Spanish sample: 164 participants (62 male and 102 female) with ages ranging from 18 and 30 (M = 21.63, SD = 2.40). In terms of education, 76.8% had completed secondary education, 17.1% had a graduate degree, 5.5% had a master’s or doctoral degree, and 0.6% had only a primary education, Furthermore, 83.5% were students, 57.9% lived with their families, and 39.6% had a partner.

Ecuador sample: 171 participants (57 males and 114 females) with ages ranging from 18 and 54 (M = 22.42, SD = 5.74). Regarding the level of education, 73.7% had secondary education, 20.5% had a graduate degree, 1.2% had a master’s or doctoral degree, and 0.6% had only a primary education. Of the respondents, 78.9% were students, 84.8% lived with their families, and 20.5% had a partner.

Peru sample: 154 participants from Peru (46 males and 108 females) with ages ranging from 18 and 46 (M = 24.74, SD = 4.75). Concerning the level of education, 25.3% had secondary education, 31.8% had a graduate degree, 3.9% had a master’s or doctoral degree and 39% had only a primary education. Moreover, 75.3% were students, 84.4% lived with their families, and 21.4% had a partner.

The full descriptive characteristics are described in Table 1.

## 6. Procedure

The data were collected between December 2021 and December 2022 through the “Qualtrics” online platform. The questionnaire was distributed in four different countries, including Italy and Spain in Europe, and Ecuador and Peru in South America. Participants were recruited online and expressed their informed consent before starting the survey. The anonymization of data was guaranteed, allowing participants to respond to the survey in any environment (e.g., at home, university, work), using PCs, smartphones, or tablets. Participants can stop or close the survey at any time, without providing explanations. Ethical approval was obtained from both the Institutional Review Board of the Comité de Ética of the University of Valencia (IRB 15910/2021) and the Institutional Review Board of the Department of Psychology, Faculty of Medicine and Psychology, “Sapienza” University of Rome (IRB 2193/2020), in accordance with the Declaration of Helsinki principles.

## 7. Materials

### 7.1. Problematic Internet Use: Self-Reported Measures

#### 7.1.1. Internet Addiction Test (IAT)

The Internet Addiction Test (IAT), developed by Young [3], is a widely used instrument for measuring addictive Internet use. It consists of 20 items based on the DSM-IV criteria, and it assesses aspects such as the fear of living without the Internet and attempts to reduce time spent online. Responses are recorded on a five-point Likert scale ranging from 1 (very rarely) to 5 (very frequently). Scores are categorized as follows: normal users or problem-free users (<40 points), and problematic Internet users (≥40 points) [53]. Both the Italian version of the IAT [54] and the Spanish version by Fernández-Villa and colleagues [55] were used in this study, with Cronbach’s alpha values of 0.85, 0.86, 0.88, and 0.90, respectively, for Italy, Spain, Ecuador, and Peru.

#### 7.1.2. Social Media Engagement Scale (SMEs)

The Social Media Engagement Scale (SMEs), developed by Przybylski and colleagues [18], assesses the frequency of social network use during daily activities. Participants rate their engagement on an eight-point Likert-type scale ranging from 1 (No day last week) to 8 (Every day last week). The scale showed high reliability, with Cronbach’s α ranging from 0.70 and 0.84.

#### 7.1.3. Bergen Social Media Addiction Scale (BSMAS)

The Bergen Social Media Addiction Scale (BSMAS) [56], is based on Griffiths’ [57] structural factor (i.e., six dimensions) scale, and consists of six items assessing aspects like salience, mood, modification, tolerance, withdrawal conflict, and relapse (e.g., “How often during the last year have you used social media so much that it has negatively impacted your job/studies?” “How often during the last year have you felt an urge to use social media more and more?”). Responses are recorded on a 5-point Likert scale. A total score of 24 or higher indicates a possible clinical diagnosis of social media addiction [48]. The Spanish version, developed by Vallejos-Flores and colleagues [58], and the Italian version, developed by Monacis and colleagues [59] were used in this study, with Cronbach’s alpha values of 0.77 (Italian sample), 0.79 (Spanish sample), 0.78 (Ecuadorian sample), 0.82 (Peruvian sample).

#### 7.1.4. Internet Gaming Disorder Scale—Short Form (IGDS9-SF)

The Internet Gaming Disorder Scale—Short Form (IGDS9-SF), developed by Pontes and Griffiths [60], assesses the severity of Internet Gaming Disorder (IGD) and its negative effects by examining both online and/or offline gaming activities (i.e., over 12 months). The scale consists of nine items, each of which corresponds to one of the nine core criteria defined by the DSM-5 [61]. Participants rate their responses on a five-point Likert scale ranging from 1 (never) to 5 (very often). Higher scores indicate a higher degree of gaming disorder.

Both the Italian version, developed by Monacis and colleagues [62], and the Spanish version, validated by Beranuy and colleagues [63], were used for this study. The Cronbach’s alpha values ranged from 0.85 and 0.93 in the four samples.

#### 7.1.5. Fear of Missing Out Scale (FoMOs)

The Fear of Missing Out Scale (FoMOs), developed by Przybylski and colleagues [18], is a 10-item self-report questionnaire designed to measure individuals’ experiences of a pervasive apprehension that others are engaged in rewarding activities and positive relationships in their absence (e.g., “I get worried when I find out my friends are having fun without me”). Respondents rate their agreement on a 5-point Likert scale ranging from 1 (not true of me) to 5 (extremely true of me). Higher scores (ranging from 1 to 5) indicate higher levels of FoMO. Both the Spanish version, developed by Gil and colleagues [64], and the Italian version, developed by Casale and Fioravanti [65], were used for this study. The Cronbach’s alpha values ranged from 0.81, 0.83, 0.83, and 0.82, for the Italian, Spanish, Ecuadorian, and Peruvian samples, respectively.

#### 7.1.6. Phubbing Scale

The Phubbing Scale (PHUB), developed by Karadag and colleagues [66], consists of ten items designed to measure Phubbing behavior on a five-point Likert scale ranging from 1 (never) to 5 (always). The scale assesses two factors: communication disturbance (e.g., “My eyes start wandering on my phone when I’m together with others”) and phone obsession (e.g., “When I wake up in the morning, I first check the messages on my phone”). Both the Italian version, developed by Guazzini and colleagues [12], and the Spanish version, validated by Blanca and Bendayan [67], were used. Cronbach’s alpha values were calculated separately for each factor. Concerning the communication disturbance factor, the alpha ranges from 0.57 to 0.80; and for the phone obsession dimension, Cronbach’s alpha ranges from 0.63 and 0.76.

### 7.2. Personality Self-Reported Measures

#### 7.2.1. Difficulties in Emotion Regulation Scale (DERS)

The Difficulties in Emotion Regulation Scale (DERS), developed by Gratz and Roemer [23], is a self-report questionnaire consisting of 36 items that assess six dimensions of difficulties in emotion regulation. These dimensions include non-acceptance, goal-directed behavior, impulse control, limited access to effective emotional regulation strategies, lack of emotional awareness, and lack of emotional clarity. In this study, the Italian short version, proposed by Lausi and colleagues [68], was used. This version consists of 20 items, and participants rated their responses on a five-point Likert scale ranging from 1 (almost never) to 5 (almost always). Higher scores indicate greater difficulties in emotion regulation. The total Cronbach’s alpha for this study was 0.88.

Additionally, for the Spanish-speaker samples, we utilized the Chilean short version of the DERS proposed by Gúzman-González and colleagues [69]. This version consists of 25 items and provides a global score, calculated as the average of all item scores, as well as scores for each scale representing different dimensions of emotional regulation (i.e., emotional rejection, emotional interference, emotional inattention, emotional lack of control and emotional confusion.). Participants responded on a 5-point Likert scale. The total Cronbach’s alpha for the Spanish sample was 0.90, for the Ecuadorian sample it was 0.90, and for the Peruvian sample it was 0.92.

#### 7.2.2. Big Five Inventory (BFI)

The Big Five Inventory-15 [70] was used in this study. This inventory was designed to assess five personality dimensions: extroversion, agreeableness, conscientiousness, neuroticism, and openness to experience. Participants rate their responses on a 5-point Likert scale, ranging from 1 (strongly disagree) to 5 (strongly agree). Item examples were: “I see myself as a person that is reserved” and “I see myself as a person that tends to find fault with others.” The adaptation of the scale for the Spanish-speaking context was conducted by Dominguez-Lara and Merino-Soto [71]. The reliability of each personality dimension, as measured by Cronbach’s alpha, ranged from 0.64 to 0.81 for extroversion, 0.67 to 0.79 for agreeableness, 0.6 to 0.67 for conscientiousness, 0.65 to 0.68 for neuroticism, and 0.53 to 0.63 for openness to experience across the Spanish-speaking samples.

In the Italian sample, the Big Five Inventory-10 (BFI-10) [72] was used. It is a self-report questionnaire assessing extroversion, agreeableness, conscientiousness, neuroticism, and openness to experience. Respondents used a 5-point Likert scale (1 = strongly disagree, 5 = strongly agree). The Italian version developed by Guido and colleagues [73], was applied. Reliability estimates for each personality dimension were 0.73 for extroversion, 0.59 for agreeableness, 0.75 for conscientiousness, 0.58 for neuroticism, and 0.74 for openness to experience.

#### 7.2.3. Prosociality Scale (PS)

The Prosociality Scale (PS), developed by Caprara and colleagues [74], is a 16-item questionnaire that assesses different behaviors and feelings associated with four types of prosocial actions: sharing, helping, taking care of, and feeling empathic towards others and their needs. Participants responded to each prosocial behavior item on a five-point Likert scale, ranging from 1 (never/almost never true) to 5 (almost always/always true). Cronbach’s alpha was 0.92.

In the case of the Spanish-speaking sample, an adapted version of the scale was used [75]. This adaptation ensures linguistic and cultural appropriateness for the Spanish-speaking participants, enhancing the validity and reliability of the instrument within this specific cultural and linguistic context. Cronbach’s alpha was 0.90, 0.91, and 0.92 for the Spanish, Ecuadorian, and Peruvian samples, respectively.

#### 7.2.4. Impulsivity Behavior Scale

The Impulsivity Behavior Scale, developed by Billeux and colleagues [76], consists of 20 items and evaluates five dimensions of impulsivity (i.e., positive urgency, negative urgency, lack of perseverance, lack of premeditation, and sensation seeking). Participants responded on a four-point Likert scale, ranging from 1 (strongly agree) to 4 (strongly disagree). Both the Italian version of the UPPS-P [77] and the Spanish version by Cándido and colleagues [78] were used in this study, with Cronbach’s alpha values of 0.85, 0.86, 0.88, and 0.90, respectively, for the Italian, Spanish, Ecuadorian, and Peruvian samples. Cronbach’s alpha for the total score was 0.71, 0.73, 0.74, and 0.79 for Italy, Spain, Ecuador, and Peru, respectively. While a Cronbach’s alpha of 0.78, 0.71, 0.56, and 0.64 for positive urgency, 0.83, 0.83, 0.75 to 0.79 for negative urgency, 0.93, 0.82, 0.73, and 0.78 for lack of perseverance, 0.82, 0.76, 0.67 to 0.80 for lack of premeditation and 0.83, 0.86 0.83 and 0.79 for sensation seeking for Italy, Spain, Ecuador, and Peru, respectively.

## 8. Data Analysis

The statistical analyses for this study were conducted using the Statistical Package for Social Science (SPSS; version 26.0; IBM SPSS, Armonk, NY, USA). Comprehensive descriptive statistics were provided for demographic variables, including age, gender, marital status, level of education, cohabitation status, and working conditions, which are reported in Table 1.

Following the descriptive phase, questionnaire scores were analyzed by using a Multivariate Analysis of Covariance (MANOVA) with (i.e., problematic Internet use, emotion, and personality self-reported measures) as dependent variables and the four countries as fixed factors. We conducted multiple comparisons using the Bonferroni correction for different dependent variables across the four countries (Italy, Spain, Ecuador, and Peru). The parametric assumptions of the equality of variance of scores between groups were checked using variance ratio tests and Levene’s tests.

Subsequently, for each country separately, partial Pearson’s correlations were conducted, followed by stepwise linear regression analyses to investigate relationships between variables and Internet addiction. Multicollinearity was assessed using the variance inflation factor (VIF) and tolerance. Statistical significance was established at *p* < 0.05. To ensure the robustness of the findings, the distributions of all data were verified for normality, and all statistical analyses were performed on de-identified data in line with ethical standards.

## 9. Results

### 9.1. Differences between Countries on Variables

#### 9.1.1. Problematic Internet Use

There was a statistically significant difference between-subjects with IAT scores (F_(3,671)_ = 11.45, *p* < 0.001, η^2^ = 0.05), in which the Italian group (*M* = 2.24; *SD* = 0.52) showed higher scores than the Spanish (*M* = 1.93, *SD* = 0.51); (*MD* = 0.32, *p* < 0.001), Ecuadorian (*M* = 2.00; *SD* = 0.56); (*MD* = 0.24, *p* < 0.001), and Peruvian groups (*M* = 2.05; *SD* = 0.56); (*MD* = 0.25, *p* < 0.001). Also, there was a statistically significant difference between-subjects of BSMAS (F_(3,671)_ = 11.58, *p* < 0.001, η^2^ = 0.05); the Italian participants (*M* = 2.01; *SD* = 0.70) reported a significantly lower score than Spanish (*M* = 2.44; *SD* = 0.72; *MD* = −0.43, *p* < 0.001), Ecuadorian (*M* = 2.29; *SD* = 0.76; *MD* = −0.29, *p* < 0.001, and Peruvian participants (*M* = 2.36; *SD* = 0.76; *MD* = −0.35, *p* < 0.001). No statistically significant differences were found between the Spanish, Ecuadorian, and Peruvian samples concerning the SMES score (*p* = 0.263). Concerning the IGDS, there was a statistically significant difference between groups (F_(3,671)_ = 3.04, *p* < 0.05, η^2^ = 0.01), in which the Italian group showed lower mean differences (*M* = 1.57; *SD* = 0.59) with respect to those of the Peruvian sample (*M* = 1.84; *SD* = 0.83; *MD* = −0.28, *p* < 0.05).

As for Phubbing, there was a statistically significant difference between groups in the communication disturbance dimension (F_(3,671)_ = 3.56, *p* < 0.05, η^2^ = 0.02), where the Italian participants showed significantly higher scores (*M* = 2.39; *SD* = 0.58) than the Peruvian ones (*M* = 2.17; *SD* = 0.71; *MD* = 0.21, *p* < 0.05). On the other hand, there are no differences among countries in the phone obsession dimension (*p* = 0.06).

As regards the FoMO, we observed statistically significant differences between groups (F_(3,671)_ = 10.47, *p* < 0.001, η^2^ = 0.05). Pairwise comparisons showed no statistically significant difference (*p* = 0.31) between the Italian (*M* = 2.51; *SD* = 0.70) and the Spanish group (*M* = 2.37; *SD* = 0.68), but Italian participants showed significantly higher scores than Ecuadorian (*M* = 2.27; *SD* = 0.71; *MD* = 0.28, *p* < 0.001), and Peruvian participants (*M* = 2.13; *SD* = 0.69; *MD* = 0.38, *p* < 0.001). Also, Spanish participants reported significantly higher scores than Peruvian participants (*M* = 2.13; *SD* = 0.69; *MD* = 0.24, *p* < 0.01). All statistical results related to the problematic use of Internet variables are shown in Table 2.

#### 9.1.2. Personological Variables

Concerning the DERS scale, there was a statistically significant difference between groups (F_(3,671)_ = 2.88, *p* < 0.05, η^2^ = 0.01), in which the Italian participants (*M* = 2.65; *SD* = 0.66) reported higher scores than the Spanish participants (*M* = 2.44; *SD* = 0.69; *MD* = 0.20, *p* < 0.05). No other significant differences were found between the mean scores of the other samples.

As for the extraversion dimension of BFI (F_(3,671)_ = 4.39, *p* < 0.01, η^2^ = 0.019), the Spanish participants showed higher scores (*M* = 3.85; *SD* = 0.92) than both the Italian (*M* = 3.52; *SD* = 0.95; *MD* = 0.34, *p* < 0.01) and Peruvian participants (*M* = 3.54; *SD* = 1.07; *MD* = 0.31, *p* < 0.05).

The neuroticism dimension showed a significant between-groups difference (F_(3,671)_ = 17.22, *p* < 0.001, η^2^ = 0.07), where the scores of the Italian sample (*M* = 3.44; *SD* = 0.91) were higher than the Spanish (*M* = 2.72; *SD* = 0.96; *MD* = −0.72, *p* < 0.001), Ecuadorian (*M* = 3.05; *SD* = 0.96; *MD* = 0.38, *p* < 0.001), and the Peruvian ones (*M* = 3.00; *SD* = 0.95; *MD* = 0.44, *p* < 0.001). In addition, the Spanish group showed lower levels of neuroticism compared to the Ecuadorian sample (*MD* = 0.33, *p* < 0.01).

As for the agreeableness dimension, there was a between-groups difference (F_(3,671)_ = 4.85, *p* < 0.005, η^2^ = 0.02), in which Italian participants (*M* = 3.44; *SD* = 0.91) showed lower scores than both the Spanish (*M* = 2.72; *SD* = 0.96; *MD* = −0.22, *p* < 0.05) and the Ecuadorian group (*M* = 2.72; *SD* = 0.96; *MD* = −0.26, *p* < 0.005).

Furthermore, a statistically significant between-groups difference was found in the openness to experience dimension (F_(3,671)_ = 6.20, *p* < 0.001, η^2^ = 0.03). The Italian sample reported lower scores (*M* = 3.52; *SD* = 0.96) than the Ecuadorian (*M* = 3.79; *SD* = 0.85; *MD* = −0.26, *p* < 0.05) and Peruvian sample (*M* = 3.84; *SD* = 0.78; *MD* = −0.32, *p* < 0.005). The same results are observed when comparing the Spanish sample (M = 3.54; SD = 0.85), with lower scores than both the Ecuadorian (*M* = 3.79; *SD* = 0.85; *MD* = −0.25, *p* < 0.05) and Peruvian samples (*M* = 3.84; *SD* = 0.78; *MD* = −0.30, *p* < 0.01). No differences between countries were found in the mean scores of the “consciousness” dimension (*p* = 0.10).

With respect to the prosocial behavior, the results showed a significant between-subjects difference (F_(3,671)_ = 5.76, *p* < 0.001, η^2^ = 0.03), in which the Italian participants showed higher scores (*M* = 3.96; *SD* = 0.65) than the Peruvian (*M* = 3.75; *SD* = 0.61; *MD* = 0.22, *p* < 0.01). Also, the Spanish sample showed higher scores (*M* = 4.01; *SD* = 0.56) than the Peruvian (*M* = 3.75; *SD* = 0.61; *MD* = 0.27, *p* < 0.001).

Finally, indicating a concern for UPPS, we observed statistically significant between-subjects difference in the lack of perseverance dimension (F_(3,671)_ = 5.42, *p* < 0.001, η^2^ = 0.02) where the Italian group reported higher scores (*M* = 2.39; *SD* = 1.01) than the Spanish (*M* = 2.10; *SD* = 0.75; *MD* = 0.29, *p* < 0.01), the Ecuadorian (*M* = 2.09; *SD* = 0.72; *MD* = 0.30, *p* < 0.005) and the Peruvian groups (*M* = 2.14; *SD* = 0.78; *MD* = 0.25, *p* < 0.05).

There was a statistically significant between-subjects difference in the negative urgency dimension (F_(3,671)_ = 2.65, *p* < 0.05, η^2^ = 0.01), in which the Ecuadorian sample reported higher scores (*M* = 3.08; *SD* = 0.91; *MD* = 0.30, *p* < 0.05) than the Peruvian sample (M = 2.78, SD = 0.91). No further differences have been observed in the positive urgency (*p* = 0.143), and lack of premeditation (*p* = 0.397), in the sensation-seeking dimension (*p* = 0.065). All statistical results related to the psychological variables are reported in Table 3.

### 9.2. Correlations between Variables among Countries

#### 9.2.1. Technological Variables

We investigated the relationships between Internet addiction scores with technological variables by using bivariate Pearson’s correlations. We conducted a separate correlation analysis for each country to investigate the presence of possible exclusive relationships. Overall, all four countries showed positive correlations between the IAT and all the other variables related to problematic Internet use (e.g., BSMAS, SMES, IGDS, Phubbing, and FoMO). All statistical results are reported in Table 4.

#### 9.2.2. Personological Variables

With respect to the personological variables, the correlation analysis showed positive correlations in all the countries between IAT and DERS, the neuroticism dimension of BFI, negative urgency, positive urgency (except for Peru), and the lack of perseverance (except for Spain) dimensions of UPPS.

The DERS, neuroticism dimension of BFI, and negative urgency dimension of UPPS-P were positively correlated with IAT among countries, while positive urgency and lack of perseverance dimensions of UPPS-P were positively correlated among countries except for Peru and Spain, respectively. With respect to the lack of premeditation dimension of UPPS-P, results showed a positive correlation with IAT for Peru and Ecuador. Concerning the extraversion, agreeableness, and open to experience dimensions of BFI and the prosociality dimension, the results showed a negative correlation with IAT for Peru only. Lastly, the consciousness dimension of BFI was negatively correlated with IAT for both Italy and Peru only. Finally, no statistically significant correlations have been observed between IAT and the sensation-seeking dimension of UPPS in the four samples. All statistical results are reported in Table 5.

### 9.3. Linear Regression Analyses

We used a stepwise linear regression analysis to evaluate which variables could significantly predict the development of the symptoms of addiction related to the use of the Internet. We conducted a separate linear regression for each country. Concerning the Italian sample (Table 6), the results yielded a model with four predictors that explained 71.4% of the variance (R_2_ = 0.714, F_(4,142)_ = 91.93, *p* < 0.001). It was found that BSMAS significantly predicted the IAT (β = 0.513, *p* < 0.001), as did IGDS (β = 0.302, *p* < 0.001), the lack of perseverance dimension of the UPPS (β = 0.181, *p* < 0.001) and lastly the phone obsession of the Phubbing scale (β = 0.169, *p* < 0.01).

Concerning the Spanish sample (Table 7), the results indicated that five predictors explained 68.6% of the variance (R_2_ = 0.686, F_(5,74)_ = 35.57, *p* < 0.001). It was found that BSMAS significantly predicted the IAT (β = 0.307, *p* < 0.001), as did IGDS (β = 0.427, *p* < 0.001), the phone obsession of Phubbing (β = 0.208, *p* < 0.01), DERS (β = 0.171, *p* < 0.05) and the lack of premeditation of UPPS (β = 0.145, *p* < 0.05).

Concerning the Ecuadorian sample (Table 8), the results showed that two predictors explained 66.7% of the variance (R_2_ = 0.667, F_(2,84)_ = 87.1, *p* < 0.001). It was found that BSMAS (β = 0.534, *p* < 0.001), and IGDS (β = 0.470, *p* < 0.001) significantly predicted the IAT.

Lastly, in the Peruvian sample (Table 9), the regression analysis reported two predictors that explained 77.5% of the variance (R_2_ = 0.775, F_(2,71)_ = 126.88, *p* < 0.001). It was found that BSMAS (β = 0.676, *p* < 0.001), and IGDS (β = 0.304, *p* < 0.001) significantly predicted the IAT.

## 10. Discussion

This study aims to investigate potential differences among countries (i.e., Italian, Spanish, Ecuadorian, and Peruvian) in developing risk, and protective factors related to problematic Internet use. The research employs a comprehensive approach, including psychological, social, and environmental factors to explore the multifaceted nature of Internet-related behaviors. By comparing data from different cultural contexts, the study offers unique patterns and commonalities that contribute to a nuanced understanding of the problematic use of the Internet.

The first research question was to investigate the presence of possible differences in technological (i.e., IA, SMA, social media engagement, IGD, Phubbing, FoMO) and personological (DERS, personality traits, prosociality, and impulsivity) variables among countries (i.e., Italy, Spain, Ecuador, and Peru).

In terms of technological variables, Italy exhibits higher scores for IA but lower scores for SMA compared to the other countries. Regarding the FoMO, Italy shows higher scores than the South American countries included in the research (i.e., Ecuador and Peru), while Spain only shows higher scores than Peru. Italy scores higher than Peru in the communication disturbance sub-dimension of Phubbing, whereas the opposite pattern is observed in IGDS. Regarding personality variables, Italy scores higher than all other countries for the sub-dimension neuroticism in the BFI-10 and the sub-dimension of lack of perseverance in the UPPS. For the sub-dimension negative urgency, Ecuador’s scores are higher than those of Peru. Only in neuroticism does the Spanish sample show lower scores than Ecuador. Italy shows lower scores in the sub-dimension agreeableness of the BFI-10, compared to Spain and Ecuador. For the sub-dimension openness to experience, both Italy and Spain show lower scores than Peru and Ecuador. Finally, in terms of extroversion, the Spanish sample demonstrates higher scores than Italy and Peru. Concerning prosociality, both Italy and Spain exhibit higher scores than Peru. In terms of the variable emotional dysregulation, Italy only scores higher than Spain.

These findings underscore the importance of considering cultural diversity in the studies of Internet-related behaviors and highlights the need for a comprehensive approach that considers the several factors influencing these behaviors. Cultural variations, as highlighted by Hofstede’s cultural dimensions theory, may influence individuals’ attitudes and behaviors toward technology use, contributing to differences in IA rates among countries [79]. For example, countries with higher levels of individualism, such as Italy and Spain, may exhibit higher scores in IA due to prioritizing personal autonomy and independence, while more collectivist cultures like Ecuador and Peru may emphasize social harmony and interconnectedness, potentially leading to lower scores in certain technological variables [80].

Numerous recent studies have underscored the significance of cultural dimensions in shaping the attitudes and behaviors related to technology use. For instance, the research by Lee and colleagues [81] emphasized the role of collectivism in influencing individuals’ psychological adjustment and Internet use patterns, highlighting potential differences between individualistic cultures like Italy and Spain and collectivist cultures like Ecuador and Peru. Additionally, findings from Van Rooij and colleagues [82] cautioned against overgeneralizing the concept of gaming disorder across different cultural contexts, suggesting that cultural factors may moderate the expression of problematic Internet behaviors.

Furthermore, individual differences in personality traits, particularly neuroticism, have been associated with higher levels of Internet addiction [83].The findings regarding higher scores in neuroticism among Italian participants align with previous research, suggesting a predisposition towards problematic Internet use in this population.

Other studies [33,84] have demonstrated the relevance of traits such as neuroticism and impulsivity in predicting susceptibility to Internet addiction. However, it is essential to interpret these findings cautiously, avoiding implications of direct causality or correlations between variables and considering the complex interplay between cultural factors and individual traits, as highlighted by Triandis [80], who emphasized the need to consider both universal and culture-specific aspects of behavior.

As per the second research question, it emerges that in all four samples, there are strong positive correlations between the IAT total score and several variables, including social media addiction, social media engagement, Internet gaming disorder, Phubbing, FoMO, and psychological traits like emotional dysregulation, neuroticism, and impulsivity due to negative urgency.

In the literature, some data exist supporting the direct relationship between Internet addiction symptoms and the maladaptive use of social media and Internet gaming [24,26,85] and, in general, with Phubbing [66].

Among these results, it is worth noting that the strong positive correlations between IAT and both the communication disturbance and phone obsession aspects of Phubbing suggest that individuals who engage in more Phubbing behavior (ignoring their partner due to phone use) during conflicts or due to an obsession with the phone are more likely to have higher scores on the IAT. This implies a cross-cultural link between problematic phone use in relationships and broader Internet addiction.

The relationship between emotional dysregulation and the problematic use of the Internet has been studied. The influence of the difficulties in emotion regulation in the development of symptoms related to the excessive use of the Internet has been confirmed in diverse cultural contexts and samples, like in a sample of Chinese adolescents [86], Italian university students [24] and Spanish university students [26]. In this line, the study has confirmed that the strong positive correlation indicates that individuals with greater difficulties in emotion regulation are more likely to exhibit higher levels of Internet addiction, as measured by the IAT. This suggests a potential relationship between challenges in regulating emotions and the development or exacerbation of Internet addiction.

The strong positive correlation implies that individuals with higher levels of neuroticism (as measured by BFI) are more likely to have higher IAT scores. This suggests a connection between certain personality traits, such as neuroticism, and the likelihood of experiencing Internet addiction.

The positive correlation between FoMO and Internet addiction across all the samples suggests that individuals who fear missing out on social experiences may be more prone to problematic Internet use. This fear might drive individuals to stay connected online constantly, contributing to higher IA scores. FoMO emerges as a pivotal determinant in the context of problematic Internet use. Individuals haunted by the apprehension of social exclusion, typified by the fear of missing out on social engagements, exhibit a pronounced correlation with IA. This phenomenon suggests a poignant interplay between the fear of being socially marginalized and the inclination to remain digitally tethered.

The strong positive correlation indicates that individuals with higher levels of negative urgency (acting impulsively under negative affect), and the positive correlation between positive urgency (UPPS) and IAT across the Italian, Spanish, and Ecuadorian samples indicates that individuals with higher levels of positive urgency (acting impulsively in response to positive emotions) are more likely to exhibit problematic Internet use. We also observe a positive correlation between IAT scores and lack of perseverance (UPPS) across the Italian, Ecuadorian, and Peruvian samples. This suggests that individuals who struggle with persistent efforts and easily abandon tasks may be more prone to problematic Internet use. These data are consistent with previous studies [50], that found that impulsiveness could be a risk factor for Internet addiction and pathological gambling.

For the Italian and Peruvian samples, a negative correlation between IAT scores and Prosocial Behavior is noticed. The negative correlation implies that lower levels of prosocial behavior (altruistic and cooperative actions) are associated with higher levels of problematic Internet use.

Regarding the relationship between personality traits and Internet addiction, we observed varying results across different samples. Specifically, in the Peruvian sample, there were negative correlations between Internet addiction and extraversion, agreeableness, conscientiousness, and openness to experience. This suggests that individuals with lower levels of these traits may be more susceptible to problematic Internet use. Similarly, in the Italian sample, we found a negative correlation between Internet addiction and conscientiousness. Conscientiousness, a personality trait associated with self-discipline, organization, goal-directed behavior, and a preference for planned over spontaneous activities [87], appears to play a significant role in mitigating Internet addiction.

In the context of problematic Internet use, individuals with lower conscientiousness may be more prone to impulsivity, lack of self-control, and difficulty in adhering to plans or schedules. This could manifest in spending excessive time on the Internet, engaging in online activities impulsively, and facing challenges in regulating Internet use. These results [87,88] individuate the consciousness trait as a protective factor in the development of Internet addiction.

The results of the stepwise multiple linear regression analysis for the Italian sample reveal significant predictors that collectively explain 71.4% of the variance in the symptoms of addiction related to Internet use (IAT). The model identifies the BSMAS as a robust predictor, indicating that higher scores on the BSMAS are associated with increased symptoms of Internet addiction. Similarly, the IGDS emerges as a significant predictor, suggesting a positive relationship between Internet gaming behavior and addiction symptoms. Moreover, the lack of premeditation and specific dimensions of impulsivity within the UPPS Impulsivity Behavior Scale contribute significantly to the prediction of Internet addiction symptoms. This indicates that individuals scoring higher on this impulsivity dimension are more likely to display symptoms of Internet addiction. Furthermore, the analysis identifies a unique contribution from the phone obsession dimension of Phubbing. This suggests that individuals who exhibit higher levels of phone obsession in their behavior are more likely to develop symptoms of Internet addiction. Finally, the goals dimension of the DERS makes a moderate yet significant contribution to the prediction model, highlighting the role of emotional regulation in the development of Internet addiction symptoms.

As for the Spanish sample, five significant predictors collectively explain a substantial portion of the variance in the symptoms of addiction related to Internet use (IAT). The model, comprising these predictors, demonstrates a strong explanatory power, accounting for 68.6% of the variance.

The analysis identifies the BSMAS as a significant predictor. Similarly, the IGDS emerges as a substantial predictor. Moreover, the phone obsession dimension of Phubbing proves to be a significant predictor. This finding suggests an association between phone-related behaviors and Internet addiction, highlighting the interconnected nature of these technological behaviors within the Spanish sample.

The analysis further reveals that the DERS demonstrates a positive association, highlighting the impact of emotional regulation on Internet addiction. Additionally, the lack of premeditation dimension of the UPPS contributes significantly to the prediction model. For the Ecuadorian and Peruvian samples, the analysis reveals that two predictors, the BSMAS and IGDS, respectively, explain 66.7% and 77.5% of the variance in IAT scores.

Despite these two samples showing comparable results, we can affirm that distinct psychological variables emerged as significant predictors of problematic Internet use in Italy, Spain, Ecuador, and Peru.

While the study provides valuable insights into problematic Internet use and the associated psychological variables across diverse cultural contexts, it is essential to recognize and acknowledge its limitations:

First, the reliance on self-report measures, such as questionnaires, introduces the possibility of social desirability bias. Participants may provide responses they believe are socially acceptable rather than reflecting their true behaviors and attitudes.

Second, despite efforts to adapt scales for linguistic and cultural appropriateness, the study assumes measurement equivalence across cultures, especially for the Spanish-speaker samples. Variations in the interpretation of survey items or cultural nuances may impact the validity of cross-cultural comparisons.

Third, no potential age-related differences in problematic Internet use were considered. Additionally, the narrow age range may limit the generalizability of findings to broader age groups.

Fourth, regional, socio-economic, and urban-rural differences within a country can influence Internet use patterns, potentially leading to an oversimplified representation of cultural factors.

Fifth, the cross-sectional nature of the study limits the ability to establish causality or determine the direction of relationships between variables. Longitudinal studies would provide more robust evidence for causal links.

Sixth, the demographic composition of the sample, with approximately 80% of participants being students. The resultant lack of heterogeneity in the sample limits the generalizability of the findings, as the perspectives and experiences of non-student populations are underrepresented. This limitation should be considered when interpreting the results, and future research should aim for a more varied sample to enhance the robustness and applicability of conclusions.

Lastly, the findings may not be easily generalized to other cultural contexts or countries not included in the study. Caution should be exercised in applying these findings to different populations. Socio-cultural, economic, and environmental factors could play a role and should be considered in future research.

Despite these limitations, the study serves as a valuable starting point for understanding the complex interplay between culture, psychological variables, and problematic Internet use. Future research should address these limitations to provide a more accurate and comprehensive understanding of the phenomenon. Specifically, future studies should incorporate longitudinal designs to establish causality, utilize a more diverse and representative sample, and consider the impact of regional and socio-economic factors within countries. Additionally, validating and adapting the measurement tools for different cultural contexts will enhance the accuracy of cross-cultural comparisons. By addressing these areas, future research can build on the findings of this study and contribute to a deeper understanding of problematic Internet use across diverse populations.

## 11. Conclusions

In an era dominated by technological advancements, understanding the nuances of Internet usage and its potential implications for mental health is of paramount im-portance. This research endeavors to unravel whether socio-cultural factors influence the development of problematic Internet use, and if there are divergent risk and protective factors among Italian, Spanish, Ecuadorian, and Peruvian samples.

In the cross-cultural analysis of technological addiction, it is quite important to con-sider that each country has its unique cultural norms and values, disparities in technological infrastructure, and Internet accessibility that could impact the prevalence and intensity of Internet use. Furthermore, cultural attitudes towards social media can vary, and individual and collective psychosocial factors, such as emotional regulation, impulsivity, and personality traits, may interact with cultural norms to shape Internet use patterns.

Understanding these contextual factors is crucial for interpreting and generalizing the study findings. It emphasizes the need for a culturally sensitive approach when addressing problematic Internet use and designing interventions tailored to the specific needs of diverse populations.

## Figures and Tables

**Table 1:** Descriptive statistics of the sample.

		**Italy**	**Spain**	**Ecuador**	**Peru**
Age	Mean	22.15	21.63	22.42	24.74
	DS	3.29	2.40	5.74	4.75
	Range	18–30	18–30	18–54	18–46
		**N (%)**			
Gender	Male	86 (46.2)	62 (37.8)	57 (33.3)	46 (29.9)
	Female	100 (53.8)	102 (62.2)	114 (66.7)	108 (70.1)
Marital status	Single	79 (42.5)	88 (53.7)	117 (68.4)	108 (70.1)
	Married	-	-	15 (8.8)	8 (5.2)
	Cohabitant	9 (4.8)	11 (6.7)	3 (1.8)	3 (1.9)
	Relationship	98 (52.7)	65 (39.6)	35 (20.5)	33 (21.4)
	Separated	-	-	1 (0.6)	1 (0.6)
	Widowed	-	-	-	1 (0.6)
Educational Level	Secondary School	5 (2.7)	1 (0.6)	1 (0.6)	60 (39.0)
	High School	124 (66.7)	126 (76.8)	126 (73.7)	39 (25.3)
	Bachelor’s degree	36 (19.4)	28 (17.1)	35 (20.5)	49 (31.8)
	Master’s degree	18 (9.7)	9 (5.5)	7 (4.1)	6 (3.9)
	Postgraduate degree	3 (1.6)	-	2 (1.2)	-
Cohabitation	Living alone	11 (5.9)	3 (1.8)	6 (3.5)	14 (9.1)
	Partner	8 (4.3)	11 (6.7)	9 (5.3)	3 (1.9)
	Partner/Children	1 (0.5)	-	10 (5.8)	5 (3.2)
	Relatives	150 (80.6)	95 (57.9)	145 (84.8)	2 (1.3)
	Cohabiting	16 (8.6)	55 (33.5)	1 (0.6)	130 (84.4)
Working Condition	Businessman	4 (2.2)	-	2 (1.2)	3 (1.9)
	Practitioner	6 (3.2)	5 (3.0)	16 (9.4)	12 (7.8)
	Manager	3 (1.6)	-	2 (1.2)	-
	Employer	1 (0.5)	-	1 (0.6)	3 (1.9)
	Manual worker	3 (1.6)	-	1 (0.6)	1 (0.6)
	Farmer	-	1 (0.6)	1 (0.6)	1 (0.6)
	Police officers	-	1 (0.6)	-	-
	Healthcare	6 (3.2)	2 (1.2)	7 (4.1)	2 (1.3)
	Teacher	2 (1.1)	5 (3.0)	3 (1.8)	10 (6.5)
	Student	150 (80.6)	137 (83.5)	135 (78.9)	116 (75.3)
	Other	11 (5.9)	13 (7.9)	3 (1.8)	6 (3.9)

**Table 2:** Differences between groups on Technological variables.

	Groups	*n*	M	SD	F	*p*	*ή* ^2^
IAT	Italian	186	2.24	0.52	11.45	0.001	0.049
	Spanish	164	1.93	0.51			
	Ecuadorian	171	2.00	0.56			
	Peruvian	154	2.00	0.60			
BSMAS	Italian	186	2.01	0.70	11.58	0.001	0.049
	Spanish	164	2.44	0.72			
	Ecuadorian	171	2.29	0.76			
	Peruvian	154	2.36	0.76			
SMES	Italian	186	3.12	0.92	1.33	0.263	0.006
	Spanish	164	3.26	0.97			
	Ecuadorian	171	3.32	0.95			
	Peruvian	154	3.23	1.03			
IGDS	Italian	147	1.57	0.59	3.04	0.030	0.014
	Spanish	80	1.68	0.64			
	Ecuadorian	87	1.77	0.82			
	Peruvian	74	1.84	0.83			
PHUB (CD)	Italian	186	2.39	0.58	3.56	0.014	0.016
	Spanish	164	2.22	0.72			
	Ecuadorian	171	2.32	0.69			
	Peruvian	154	2.17	0.71			
PHUB (OBS)	Italian	186	3.08	0.64	2.48	0.060	0.011
	Spanish	164	3.17	0.62			
	Ecuadorian	171	3.23	0.78			
	Peruvian	154	3.03	0.77			
FoMO	Italian	186	2.51	0.70	10.47	0.001	0.045
	Spanish	164	2.37	0.68			
	Ecuadorian	171	2.27	0.71			
	Peruvian	154	2.13	0.69			

**Table 3:** Differences between groups on Personological variables.

	Groups	*n*	M	SD	F	*p*	*ή* ^2^
DERS	Italian	186	2.65	0.66	2.88	0.035	0.013
	Spanish	164	2.44	0.69			
	Ecuadorian	171	2.57	0.80			
	Peruvian	154	2.48	0.68			
BFI (E)	Italian	186	3.52	0.95	4.38	0.005	0.019
	Spanish	164	3.85	0.92			
	Ecuadorian	171	3.58	0.87			
	Peruvian	154	3.54	1.07			
BFI (N)	Italian	186	3.44	0.91	17.22	0.001	0.071
	Spanish	164	2.72	0.96			
	Ecuadorian	171	3.05	0.96			
	Peruvian	154	3.00	0.95			
BFI (A)	Italian	147	4.06	0.73	4.85	0.002	0.021
	Spanish	80	4.27	0.61			
	Ecuadorian	87	4.31	0.67			
	Peruvian	74	4.23	0.74			
BFI (C)	Italian	186	3.98	0.90	2.11	0.098	0.009
	Spanish	164	3.97	0.67			
	Ecuadorian	171	4.14	0.71			
	Peruvian	154	4.11	0.73			
BFI (O)	Italian	186	3.52	0.96	6.20	0.001	0.027
	Spanish	164	3.54	0.85			
	Ecuadorian	171	3.79	0.85			
	Peruvian	154	3.84	0.78			
PS	Italian	186	3.96	0.65	5.76	0.001	0.025
	Spanish	164	4.01	0.56			
	Ecuadorian	171	3.89	0.61			
	Peruvian	154	3.75	0.61			
UPPS (NU)	Italian	186	2.95	1.00	2.65	0.048	0.012
	Spanish	164	2.87	1.13			
	Ecuadorian	171	3.08	0.91			
	Peruvian	154	2.78	0.91			
UPPS (PU)	Italian	186	2.89	0.87	1.81	0.143	0.008
	Spanish	164	2.95	0.84			
	Ecuadorian	171	3.09	0.78			
	Peruvian	154	2.99	0.75			
UPPS (LPr)	Italian	186	2.16	0.77	0.99	0.397	0.004
	Spanish	164	2.27	0.75			
	Ecuadorian	171	2.25	0.70			
	Peruvian	154	2.17	0.77			
UPPS (LPe)	Italian	186	2.39	1.01	5.42	0.001	0.024
	Spanish	164	2.10	0.75			
	Ecuadorian	171	2.09	0.72			
	Peruvian	154	2.14	0.78			
UPPS (SS)	Italian	186	3.07	0.89	2.42	0.065	0.011
	Spanish	164	3.15	0.98			
	Ecuadorian	171	3.30	0.95			
	Peruvian	154	3.19	0.84			

**Table 4:** Correlation coefficients (Pearson’s r)—Technological variables.

	BSMAS	SMES	IGDS	PHUB (CD)	PHUB (OBS)	FoMO
**Italy**						
IAT	0.750 **	0.242 **	0.571 **	0.442 **	0.523 **	0.464 **
N	186	186	147	186	186	186
**Spain**						
IAT	0.735 **	0.296 **	0.633 **	0.483 **	0.542 **	0.515 **
N	164	164	80	164	164	164
**Ecuador**						
IAT	0.738 **	0.318 **	0.649 **	0.401 **	0.542 **	0.430 **
N	171	171	87	171	171	171
**Peru**						
IAT	0.763 **	0.380 **	0.686 **	0.469 **	0.566 **	0.560 **
N	154	154	74	154	154	154

Note. ** *p* < 0.001; BSMAS = Bergen Social Media Addiction Scale; SMES = Social Media Engagement Scale; IGDS = Internet Gaming Disorder Scale; PHUB (CD) = Phubbing—Communication Disturbance; PHUB (OBS) = Phubbing—Phone Obsession; FoMO = Fear of Missing Out.

**Table 5:** Correlation coefficients (Pearson’s r)—Personological variables.

	DERS	BFI (E)	BFI (N)	BFI (A)	BFI (C)	BFI (O)	PS	UPPS (NU)	UPPS (PU)	UPPS (LPr)	UPPS (LPe)	UPPS (SS)
**Italy**												
IAT	0.426 **	−0.122	0.386 **	−0.020	−0.332 **	0.055	0.062	0.247 **	0.262 **	0.017	0.335 **	−0.013
N	186	186	186	186	186	186	186	186	186	186	186	186
**Spain**												
IAT	0.435 **	−0.065	0.278 **	−0.129	−0.065	−0.085	−0.080	0.361 **	0.230 **	0.101	0.135	0.014
N	164	164	164	164	164	164	164	164	164	164	164	164
**Ecuador**												
IAT	0.310 **	−0.086	0.298 **	−0.080	−0.119	−0.034	0.032	0.302 **	0.245 **	0.157 *	0.195 *	0.100
N	171	171	171	171	171	171	171	171	171	171	171	171
**Peru**												
IAT	0.458 **	−0.359 **	0.193 *	−0.365 **	−0.325 **	−0.258 **	−0.287 **	0.299 **	0.145	0.345 **	0.259 **	−0.066
N	154	154	154	154	154	154	154	154	154	154	154	154

Note. * *p* < 0.05, ** *p* < 0.01; DERS = Difficulties in Emotion Regulation Scale; BFI (E) = Big Five Inventory (Extraversion); BFI (N) = Big Five Inventory (Neuroticism); BFI (A) = Big Five Inventory (Agreeableness); BFI (C) = Big Five Inventory (Conscientiousness); BFI (O) = Big Five Inventory (Openness to Experience); PS = Prosociality Scale; UPPS (NU) = Impulsivity Behavior Scale (Negative Urgency); UPPS (PU) = Impulsivity Behavior Scale (Positive Urgency); UPPS (LPr) = Impulsivity Behavior Scale (Lack of Premeditation); UPPS (LPe) = Impulsivity Behavior Scale (Lack of Perseverance); UPPS (SS) = Impulsivity Behavior Scale (Sensation Seeking).

**Table 6:** Multiple linear regression of IAT predictors in the Italian sample.

	B	SE B	β	*p*	VIF
(Constant)	0.441	0.125	-	0.001	-
BSMAS	0.379	0.043	0.513	0.001	1.76
IGDS	0.270	0.044	0.302	0.000	1.24
UPPS (LPe)	0.096	0.025	0.181	0.000	1.16
PHUB (OBS)	0.133	0.069	0.169	0.010	1.63
R_2_ = 0.714, F_(4,142)_ = 91.93, *p* < 0.001					

Note. BSMAS = Bergen Social Media Scale; IGDS = Internet Gaming Disorder Scale—Short Form; UPPS (LPe) = Impulsivity Behavior Scale (Lack of Perseverance); PHUB (OBS) = Phubbing—Phone Obsession.

**Table 7:** Multiple linear regression of IAT Total Score predictors in the Spanish sample.

	B	SE B	β	*p*	VIF
(Constant)	−0.415	0.234	-	0.080	-
BSMAS	0.235	0.069	0.307	0.001	2.02
IGDS	0.392	0.064	0.427	0.000	1.21
PHUB (OBS)	0.183	0.069	0.208	0.010	1.56
DERS	0.149	0.061	0.171	0.017	1.24
UPPS (LPr)	0.116	0.052	0.145	0.027	1.04
R_2_ = 0.686, F_(5,74)_ = 35.57, *p* < 0.001					

Note. BSMAS = Bergen Social Media Scale; IGDS = Internet Gaming Disorder Scale—Short Form; PHUB (OBS) = Phubbing—Phone Obsession UPPS (LPr) = Impulsivity Behavior Scale (Lack of Premeditation).

**Table 8:** Multiple linear regression of IAT Total Score predictors in the Ecuadorian sample.

9	B	SE B	β	*p*	VIF
(Constant)	0.463	0.131	-	0.001	-
BMSA_TOT	0.424	0.052	0.534	0.000	1.27
IGDS_TOT	0.354	0.050	0.470	0.000	1.27
R_2_ = 0.667, F_(2,84)_ = 87.1, *p* < 0.001					

Note. IGDS = Internet Gaming Disorder Scale—Short Form.

**Table 9:** Multiple linear regression of IAT Total Score predictors in the Peruvian sample.

	B	SE B	β	*p*	VIF
(Constant)	0.322	0.117	-	0.007	-
BMSA_TOT	0.564	0.056	0.676	0.000	1.47
IGDS_TOT	0.244	0.054	0.304	0.000	1.47
R_2_ = 0.775, F_(2,71)_ = 126.88, *p* < 0.001					

Note. IGDS = Internet Gaming Disorder Scale—Short Form.

## Data Availability

Data will be made available on reasonable request.
